# Frequency of cardioversions as an additional risk factor for stroke in atrial fibrillation – the FinCV-4 study

**DOI:** 10.1080/07853890.2022.2077430

**Published:** 2022-05-20

**Authors:** Samuli Jaakkola, Tuomas O. Kiviniemi, Jussi Jaakkola, Jussi-Pekka Pouru, Ilpo Nuotio, Tuija Vasankari, Juha E. K. Hartikainen, K. E. Juhani Airaksinen

**Affiliations:** a Heart Center, Turku University Hospital and University of Turku, Turku, Finland; b Cardiovascular Medicine, Brigham and Women’s Hospital, Harvard Medical School, Boston, MA, USA; c Heart Center, Satakunta Central Hospital, Pori, Finland; d Department of Acute Internal Medicine, Turku University Hospital and University of Turku, Turku, Finland; e Heart Center, Kuopio University Hospital, Kuopio, Finland

**Keywords:** Atrial fibrillation, stroke, cardioversion

## Abstract

**Background:**

Patients with atrial fibrillation (AF) are selected for oral anticoagulation based on individual patient characteristics. There is little information on how clinical AF burden associates with the risk of ischaemic stroke or systemic embolism (SSE). The aim of this study was to explore the association of the frequency of cardioversions (CV) as a measure of clinical AF burden on the long-term SSE risk, with a focus on patients at intermediate stroke risk based on CHA_2_DS_2_-VASc score. For these patients, additional SSE risk stratification by assessing CV frequency may aid in the decision on whether to initiate oral anticoagulation.

**Methods:**

This retrospective analysis of FinCV Study from years 2003–2010 included 2074 patients who were not using any oral anticoagulation (long term or temporary) after CVs and undergoing a total of 6534 CVs for AF from emergency departments of three hospitals. Two study groups were formed: high CV frequency (mean interval between CVs ≤12 months and low frequency (>12 months).

**Results:**

A total of 107 SSEs occurred during a mean follow-up of 5.4 years. The event rates per 100 patient-years were 1.82 and 0.67 in high versus low CV frequency groups, respectively. After adjustment for CHA_2_DS_2_-VASc score, CV frequency independently predicted SSE (HR, 2.87 [95% CI, 1.47 to 5.64]; *p* = .002) at 3 years. Competing risk analysis also identified CV frequency (sHR, 2.70 [95% CI, 1.38–5.31]; *p* = .004) as an independent predictor for SSE. In patients with CHA_2_DS_2_-VASc score 1 and low CV frequency, the SSE risk was only 0.08 per 100 patient-years.

**Conclusions:**

Frequency of CVs for symptomatic AF episodes provides additional information on stroke risk in AF patients with CHA_2_DS_2_-VASc score 1.

**Key messages**
- This retrospective study offers a unique opportunity to observe the natural course of AF patients with infrequent episodes of clinical arrhythmia when they were not using OAC (before introduction of CHA_2_DS_2_-VASc score).
- Stroke or systemic embolism rate was very low (0.08 per 100 patient-years) in patients with one CHA_2_DS_2_-VASc point who visited the emergency room for cardioversion less than once a year.
- Frequency of cardioversions can be used for additional risk stratification in patients at intermediate risk of stroke based on CHA_2_DS_2_-VASc score.

## Introduction

Patients with atrial fibrillation (AF) are selected for oral anticoagulation (OAC) therapy based on individual clinical characteristics using the CHA_2_DS_2_-VASc score [1]. The pattern and characteristics of AF, however, are currently not included in stroke risk stratification schemes. Consequently, indications for OAC are similar for patients with rarely occurring short AF paroxysms and patients with permanent/persistent AF regardless of the higher risk of thromboembolic complications in the latter form of AF manifestation [2]. Accumulating scientific evidence suggests that paroxysmal AF burden (i.e. both frequency and duration of AF paroxysms) can be considered as an independent risk factor for cardiogenic stroke [3,4]. However, data on total AF burden (i.e. symptomatic and asymptomatic episodes) are obtained from patients with cardiovascular implantable electronic devices (CIED) limiting the implementation of these results to clinical management of AF. So far, there are no studies evaluating the influence of clinical AF burden measured by the frequency of symptomatic AF episodes on thromboembolic complications. Thus, the risk of stroke and systemic embolism (SSE), and the safety and efficacy of OAC therapy in patients with infrequent symptomatic AF episodes is yet to be explored. We set to perform a retrospective analysis on the risk of thromboembolic complications based on the frequency of cardioversions (CV) for acute AF in patients without OAC using the large FinCV study database [5].

## Methods

**Figure 1.**
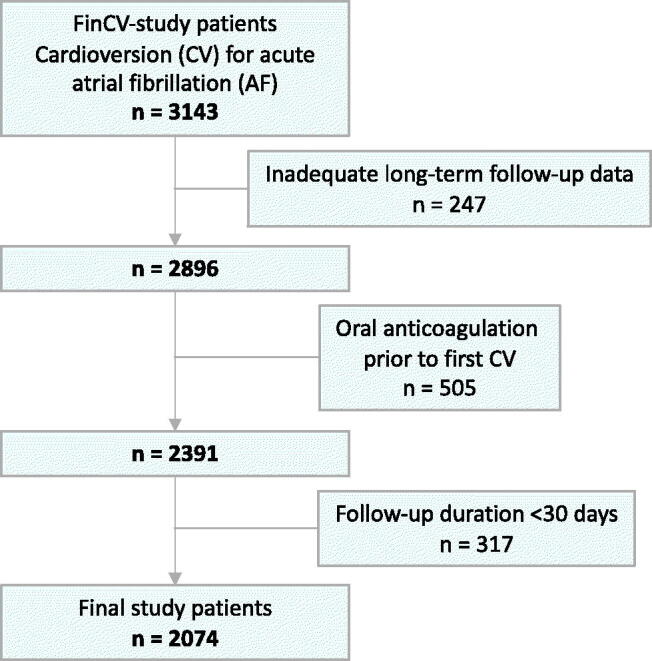
Patient selection flow chart.

This study is a part of the multicenter FinCV study (ClinicalTrials.gov Identifier: NCT01380574) assessing the thromboembolic complications after CV of acute (<48 h) AF [5,6]. Patient selection for FinCV study has been previously described in detail [5]. In short, all patients with AF diagnosis (ICD-10 code I48), age >18 years and CV for acute (<48 h) AF episode in three Finnish hospitals were identified. The AF diagnosis was based on a 12-lead electrocardiogram. Of the identified cohort, only those living within the hospital catchment area were included to ensure reliable follow-up data. In total, 3143 patients who underwent 7660 cardioversions between 2003 and 2010 were identified. For the current analysis, we included 2074 patients who were not using long-term (>4 weeks) OAC after CV and having long-term follow-up data in their patient records (Figure 1) with no patients lost to follow-up. Follow-up data were collected until any of the following events: AF was deemed permanent, initiation of long-term (>4 weeks) OAC therapy or death. If there were no end-point events, the follow-up ended on the date of the last entry in the patient record with no OAC in the medication. To assess the long-term SSE risk of patients and to exclude CV-related SSEs, the early periods of 30 days after CVs were censored from the analyses. Electronic patient records were reviewed manually following a standardised protocol, and the data were collected and managed using REDCap electronic data capture tools hosted at Turku University [7]. All baseline characteristics, thromboembolic complications, number of CVs, mortality data and anticoagulation status were recorded. SSE was defined as an ischaemic stroke documented clinically and confirmed by computerised tomography or magnetic resonance imaging or a systemic embolism confirmed by imaging, surgery, or autopsy. Data on the date and cause of death were obtained from Statistics Finland, a governmental agency which reviews all death certificates issued in Finland.

The CHA_2_DS_2_-VASc score points were calculated according to the 2019 American College of Cardiology/American Heart Association Task Force on Clinical Practice Guidelines and the Heart Rhythm Society (score point for female sex only in patients with age >65 years or ≥2 other score points) [8].

Two study groups were formed according to the frequency of CVs during the follow-up: high CV frequency (mean interval between CVs ≤12 months) and low frequency (interval >12 months). The cut-off interval of 12 months was selected based on clinical experience as there are no previous studies investigating the association between CV frequency as an SSE.

The study protocol was approved by the Medical Ethics Committee of the Hospital District of Southwest Finland and the ethics committee of the National Institute for Health and Welfare. Informed consent was not required due to the retrospective study setting. The study complies with the Declaration of Helsinki as revised in 2013.

Statistical analyses were performed using SPSS v. 27.0 statistical software (IBM Corporation, New York, USA) and Stata/SE 16.1 for Mac (StataCorp, TX, USA). Continuous variables were reported as mean ± standard deviation (SD) if normally distributed, and as median and interquartile range (IQR) if skewed. The data were tested for normal distribution using Kolmogorov–Smirnov and Shapiro–Wilk tests. Categorical variables were described as counts and percentages. Cox proportional hazards model, unpaired t-test, and Mann–Whitney test were used for univariable analysis as appropriate. The Cox regression proportional hazards assumption was assessed using graphical methods. Multivariable Cox regression analyses were used to analyse the association of CV frequency and SSE and death. Cause-specific competing risk subdistribution hazard (sHR) models accounting for death were used for SSE analysis. Cumulative incidence function, person-years and linearised rate analysis were used to estimate the cumulative SSE rate after index CV. All tests were 2-sided and *p*-value <.05 was considered statistically significant.

## Results

The median CV interval was 40.8 (IQR 21.9 − 88.7) months in the low CV frequency and 5.3 (IQR 2.9 − 8.3) months in the high frequency group. Patients in the high CV group had more co-morbidities and higher CHA_2_DS_2_-VASc score than patients with low CV frequency (Table 1). Accordingly, the median follow-up time with no oral anticoagulation use was shorter 1.0 years (IQR 0.5–2.6 years) in the high CV-frequency group compared with a median follow-up of 8.9 years (IQR 4.5–12.2 years) in the low CV-frequency group.

**Table 1.** Patient characteristics.

Characteristics	High CV Frequency (*n* = 663)	low CV frequency (*n* = 1411)	*p* Value
Age, median (IQR), y	66 (58–73)	59 (50–68)	<.001
Female, No. (%)	262 (39.5)	459 (32.5)	.002
Heart failure, No. (%)	38 (5.7)	26 (1.8)	<.001
Hypertension, No. (%)	353 (53.2)	562 (39.8)	<.001
Diabetes mellitus, No. (%)	72 (10.9)	99 (7.0)	.004
History of ischaemic stroke / TIA, No. (%)	37 (5.6)	51 (3.6)	.047
Peripheral vascular disease, No. (%)	77 (11.6)	75 (5.3)	<.001
History of myocardial infarction, No. (%)	64 (9.65)	77 (5.5)	.001
Chronic kidney disease, No. (%)	13 (2.0)	11 (0.8)	.026
Pacemaker, No. (%)	19 (2.9)	18 (1.3)	.019
Follow-up duration, median (IQR), years	1.01 (0.48-2.57)	8.87 (0.48-2.59)	<.001
CHA_2_DS_2_-VASc at FU end, mean (SD)	2.65 (1.74)	2.27 (1.73)	<.001
CHADS_2_ at FU end, mean (SD)	1.22 (1.05)	1.10 (1.09)	.015

*Abbreviations*: CHA_2_DS_2_-VASc: congestive heart failure, hypertension, age ≥75 (doubled), diabetes mellitus, and prior stroke, transient ischaemic attack or thromboembolism (doubled), vascular disease, age 65–74, sex category (female); CV: cardioversion; FU: follow-up; IQR: interquartile range; TIA: transient ischaemic attack. CHADS_2_: congestive heart failure, hypertension, age ≥75, diabetes mellitus and prior stroke, transient ischaemic attack or thromboembolism (doubled). High cardioversion frequency = mean CV interval ≤12 months, low >12 months.

A total of 107 (5.2% of patients) SSEs (97 ischaemic strokes and 10 peripheral embolisms) occurred during the median follow-up of 5.4 years (IQR 1.9–10.6 years) when the patients were not using OAC. There were 27 SSEs in the high CV frequency group (*n* = 663) and 80 SSEs in the low CV frequency group (*n* = 1411) and the linearised SSE rates per 100 patient-years were 1.82 and 0.67, respectively. In addition, the transient ischaemic attack rates per 100 patient years were 0.74 (*n* = 11) in the high and 0.24 (*n* = 29) in the low CV frequency groups.

**Figure 2.**
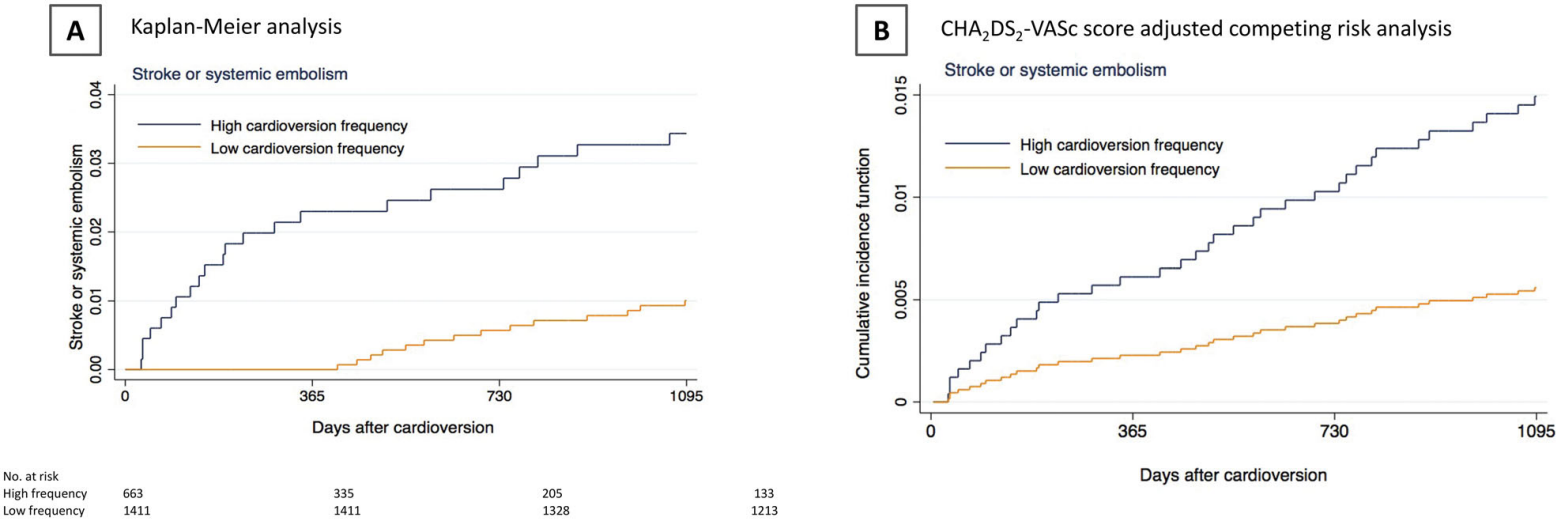
Cumulative incidence of stroke or systemic embolism (Panel A) and CHA_2_DS_2_-VASc score-adjusted competing risk analysis of stroke or systemic embolism rate (Panel B). High cardioversion frequency: mean CV interval ≤12 months, Low >12 months. *Abbreviations*: CHA_2_DS_2_-VASc, Congestive heart failure, hypertension, age ≥75 (doubled), diabetes mellitus, and prior stroke, transient ischaemic attack or thromboembolism (doubled), vascular disease, age 65–74, Sex category (female).

**Figure 3.**
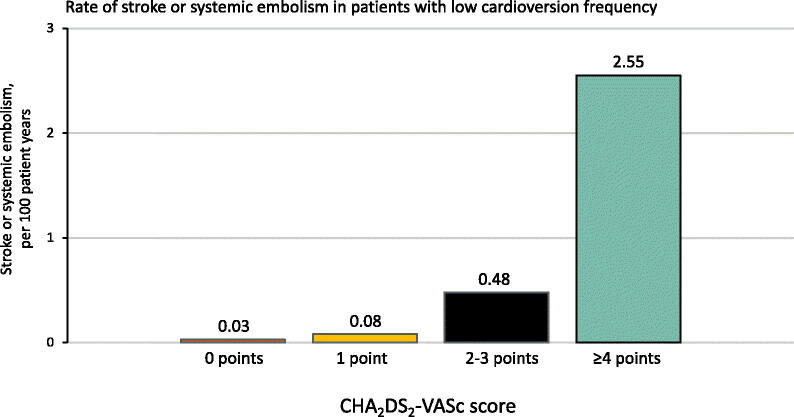
Rate of stroke or systemic embolism in patients with low cardioversion frequency. Low cardioversion frequency: mean CV interval >12 months. *Abbreviations*: CHA_2_DS_2_-VASc: Congestive heart failure, hypertension, age ≥75 (doubled), diabetes mellitus, and prior stroke, transient ischaemic attack or thromboembolism (doubled), vascular disease, age 65–74, sex category (female).

Kaplan-Meier analysis of the cumulative incidence of SSE is presented in Figure 2, Panel A. Of the 80 SSEs in patients with low CV frequency, only 1 occurred in patients with low SSE risk (CHA_2_DS_2_-VASc score 0) and 2 in those with intermediate SSE risk (CHA_2_DS_2_-VASc score 1) yielding the SSE rates of 0.03 and 0.08 per 100 patient-years, respectively (Figure 3). On the other hand, in the low CV frequency group a total of 69 SSEs occurred in patients with CHA_2_DS_2_-VASc ≥4, yielding 2.55 SSEs per 100 patient years. Cox regression analysis revealed CV frequency as an independent predictor for SSE (HR, 2.87 [95% CI, 1.47–5.64]; *p* = .002) at 3 years after CHA_2_DS_2_-VASc score adjustment. Competing risk analysis identified that both CV frequency (sHR, 2.70 [95% CI, 1.38–5.31]; *p* = .004) and increasing CHA_2_DS_2_-VASc score (sHR, 1.87 [95% CI, 1.66–2.11]; *p* <.001) were independent predictors for SSE at 3 years (Figure 2, Panel B).

There were 170 deaths during the study follow-up, 12 (7.0%) of which occurred within 30 days of SSE. All-cause mortality per 100 patient-years was 9.0 in the high CV-frequency group and 1.4 in the low-frequency group. Cox regression analysis confirmed CV frequency (HR, 2.54 [95% CI, 1.73–3.74]; *p* < .001) and CHA_2_DS_2_-VASc score (HR, 1.50 [95% CI, 1.39–1.61]; *p* < .001) as independent predictors for mortality. The most common causes of death were malignancies (24.1%), infections (12.9%), heart failure (8.8%) and acute coronary syndrome (8.2%) (Supplementary Table 1).

## Discussion

The large FinCV database from years 2003 to 2010 (before introduction of CHA_2_DS_2_-VASc score) provided us a unique opportunity to assess the natural course of AF in patients with infrequent episodes of clinical arrhythmia when they were not using OAC according to the current guideline recommendations [5,6]. Our findings show a dose-dependent association between CV frequency and SSEs and all-cause mortality in non-anticoagulated patients with paroxysmal AF. These results suggest that the effect of AF burden as an additional risk factor for thromboembolic complications can be estimated based on the quantity of symptomatic AF episodes in this selected patient group visiting emergency department. The predictive ability of CV frequency was independent of clinical patient characteristics, which are currently used for SSE risk stratification, that is, the guideline recommended CHA_2_DS_2_-VASc score.

Currently, data on symptomatic AF burden as an SSE risk factor is limited to the finding that non-paroxysmal manifestation of AF increases the SSE risk when compared to paroxysmal AF [2]. When assessing the SSE risk within the entity of paroxysmal AF, the only available data is derived from patients with CIEDs or ECG patches [4,9]. A retrospective cohort study including 1965 patients without anticoagulation (mean CHA_2_DS_2_-VASc score 2.6) explored the association between the incidence of SSE and AF burden using a 14-day ECG patch recording and found an unadjusted SSE rate of 1.51 per 100 person-years [4] in the overall study cohort. Importantly, SSE incidence increased dose-dependently (CHA_2_DS_2_-VASc score adjusted HR 3.16) when the patients in the highest and lowest AF burden tertiles were compared (0.8 in the lowest burden tertile and 2.9 in the highest). Similar increase in the stroke risk was reported in the ASSERT study when assessing the association between the duration of CIED detected subclinical AF episodes and clinical outcomes [9]. The investigators found a 3.24 (95% CI, 1.51–6.95; *p* = .003) adjusted HR for SSE in patients with AF episode durations ≥24 h, and a 3.08 SSE rate per 100 patient years. In our present study, we found a similar adjusted HR for SSE (2.9) when symptomatic AF paroxysms led to CVs more than once a year (event rate 1.82 per 100 patient years) as compared to less frequent CVs. The effect of CV frequency on SSE risk remained significant (HR 2.68 at 3 years) also in the competing risk analysis. These results suggest that increasing number of CVs for symptomatic AF episodes can be used for stroke risk stratification analogously to device collected AF burden.

Assessment of AF burden as an additional risk factor for SSE may be helpful in patients with intermediate thromboembolic risk, that is, CHA_2_DS_2_-VASc score 1 and/or in those with high risk of bleeding complications. In patients with high score, the SSE risk is high enough to justify anticoagulation regardless of AF burden [1,3]. Our results support this concept as those with CHA_2_DS_2_-VASc ≥4 and low CV frequency had a SSE rate of 2.55 exceeding the generally accepted threshold of OAC initiation (1.2 − 2.3 per 100 person years) [1,10]. At present, there is no clear consensus on anticoagulation of patients with CHA_2_DS_2_-VASc score 1. Therefore, additional thromboembolic risk factor evaluation as well as careful bleeding risk assessment and patient preference are recommended for decision-making [11]. The most important finding in our study was that the SSE rate was very low (0.08 per 100 patient-years) in patients with only one CHA_2_DS_2_-VASc point who visited the emergency room for CV less than once a year. Because the SSE risk of these patients is classified as intermediate according to the patient characteristics, the frequency of CVs seems useful in further stratifying the individual SSE risk and selecting patients for OAC therapy. In the low CV frequency group, there was a steep (over a 30-fold) increase in the SSE risk between patients with CHA_2_DS_2_-VASc 0-1 and ≥4 (Figure 3) compared with the corresponding risk increase in the CHA_2_DS_2_-VASc score validation data [12]. This finding highlights the low SSE risk in patients with low clinical AF burden and low or intermediate risk based on CHA_2_DS_2_-VASc score.

The finding that CV frequency-based arrhythmia burden can be used as a prognostic marker is consistent with earlier studies [13]. However, comparing the risk between different studies is challenging considering the fact that our study is the only one evaluating symptomatic paroxysmal AF burden in patients without CIEDs. Recent meta-analysis by Ganesan et al. exploring the outcomes of patients with non-paroxysmal AF versus paroxysmal AF, found an association between increasing amount of AF and mortality^2^. The investigators reported a 1.22 (95% CI, 1.09–1.37; *p* < .001) adjusted HR for all-cause mortality in patients with non-paroxysmal AF versus paroxysmal AF [2]. Furthermore, in a case-crossover study of 3131 CIED patients, a significant association between AF progression to longer episode durations and all-cause mortality was reported [13]. Similarly, a recent study reported that persistent/permanent AF associated with larger left atrial diameter (than in paroxysmal AF) which in turn associated with major cardiac events [14]. In our current study, the frequency of CVs predicted all-cause mortality with an adjusted HR of 2.54 for those with frequent CVs. Recent randomised trials have suggested that rhythm control therapy for symptomatic patients is beneficial in terms of patient outcome, especially as an early treatment strategy [15,16]. While AF is a progressing disease, our findings of worse outcome with frequent symptomatic AF episodes further indirectly support active upstream treatment.

The limitations of a retrospective study apply also to this study. In addition, our results apply only for AF patients who visit emergency department because of acute symptomatic AF episodes and are treated with CV. As expected, the follow-up was short in patients with frequent CVs, since long-term OAC therapy was often initiated during repeated hospital visits. On the other hand, patients with infrequent CVs – the main target group of our study – had a median follow-up of 9 years. Considering the current knowledge and guidelines (this study was conducted before the contemporary guideline recommendations on OAC for AF) it is challenging to get this kind of data on long-term natural course of this patient group. Even though subclinical AF episodes may have occurred in these patients during the long follow-up, our results show a very low SSE risk in those with low symptomatic AF burden (i.e. infrequent need of CVs) and CHA_2_DS_2_-VASc score 0–1. Important strength of our study is the Finnish health care system with comprehensive coverage of electronic patient records allowing reliable follow-up data on all study patients. As the cause of death was not assigned as a predetermined study question, the explanation for malignancies being the most common cause of death will remain hypothetical. It may well be merely coincidental, but for example a malignancy associated contraindication to OAC such as a high bleeding risk may be linked to the high number of malignancy-related deaths.

In conclusion, this study is the first to describe a significant association between symptomatic paroxysmal AF burden and clinical outcome in patients without CIEDs. Both adjusted Cox regression and competing risk analysis revealed CV frequency to be a strong independent predictor of both SSE and all-cause mortality during three-year follow-up. Most importantly, patients at intermediate SSE risk based on CHA_2_DS_2_-VASc score 1 and infrequent symptomatic AF attacks had a very low SSE risk which is a clinically useful finding when considering OAC therapy for these patients.

## Supplementary Material

Supplemental Material

## Data Availability

The data that support the findings of this study are available from the corresponding author [SJ], upon reasonable request.
