# Supplementary material for: Frequency of cardioversions as an additional risk factor for stroke in atrial fibrillation – the FinCV-4 study
Source: Ann Med. 2022 May 20;54(1):1452–8. doi: 10.1080/07853890.2022.2077430 (PMC9132398; doi:10.1080/07853890.2022.2077430)
Supplement: Supplemental Material [file IANN_A_2077430_SM5591.docx]

Supplementary Table 1. Causes of death.

|  | **n (%)** |
| --- | --- |
| Malignancy | 41 (24.1) |
| Infections | 22 (12.9) |
| Heart failure | 15 (8.82) |
| Acute coronary syndrome | 14 (8.23) |
| Gastrointestinal | 11 (6.47) |
| Ischemic stroke | 11 (6.47) |
| Accidents | 10 (5.88) |
| Other | 10 (5.88) |
| Dementia | 6 (3.53) |
| Other cardiovascular | 6 (3.53) |
| Respiratory diseases | 5 (2.94) |
| Hemorrhagic stroke | 4 (2.35) |
| Missing | 15 (8.82) |
